# Construction and verification of aggressive behavior risk prediction model in stable patients with schizophrenia

**DOI:** 10.1186/s12888-023-05296-5

**Published:** 2023-11-02

**Authors:** Yujing Sun, Wenlong Jiang, Hong Yu, Jing Zhang, Yuqiu Zhou, Fei Yin, Hong Su, Yannan Jia

**Affiliations:** 1 Department of Nursing, Harbin Medical University Daqing Campus, Daqing, China; 2 The Fourth Affiliated Hospital of Harbin Medical University, Harbin, China; 3 Daqing Third Hospital, Daqing, China

**Keywords:** Schizophrenia, Aggressive behavior, Nomogram, Prediction model

## Abstract

**Background:**

Among all types of mental disorders, individuals with schizophrenia exhibit the highest frequency of aggressive behavior. This disrupts the healthcare environment and poses threats to family life and social harmony. Present approaches fail to identify individuals with schizophrenia who are predisposed to aggressive behavior. In this study, we aimed to construct a risk prediction model for aggressive behavior in stable patients with schizophrenia, which may facilitate early identification of patients who are predisposed to aggression by assessing relevant factors, enabling the management of high-risk groups to mitigate and prevent aggressive behavior.

**Methods:**

A convenience sample of stable inpatients with schizophrenia were selected from Daqing Municipal Third Hospital and Chifeng Municipal Anding Hospital from March 2021 to July 2023. A total of 429 patients with stable schizophrenia who met the inclusion criteria were included. A survey was conducted with them using a questionnaire consisting of general information questionnaire, Positive and Negative Symptom Scale, Childhood Trauma Questionnaire-Short Form, Connor-Davidson Resilience Scale and Self-esteem Scale. Patients enrolled in this study were divided into aggressive and non-aggressive groups based on whether there was at least one obvious and recorded personal attack episode (including obvious wounding and self-injurious behavior) following diagnosis. Binary Logistic regression was used to determine the influencing factors, and R software was used to establish a nomogram model for predicting the risk of aggressive behavior. Bootstrap method was used for internal validation of the model, and the validation group was used for external validation. C statistic and calibration curve were used to evaluate the prediction performance of the model.

**Results:**

The model variables included Age, Duration of disease, Positive symptom, Childhood Trauma, Self-esteem and Resilience. The AUROC of the model was 0.790 (95% CI:0.729–0.851), the best cutoff value was 0.308; the sensitivity was 70.0%; the specificity was 81.4%; The C statistics of internal and external validation were 0.759 (95%CI:0.725–0.814) and 0.819 (95%CI:0.733–0.904), respectively; calibration curve and Brier score showed good fit.

**Conclusions:**

The prediction model has a good degree of discrimination and calibration, which can intuitively and easily screen the high risk of aggressive behavior in stable patients with schizophrenia, and provide references for early screening and intervention.

## Background

Aggressive behavior in patients with schizophrenia refers to destructive behaviors exhibited by them, including verbal and physical attacks on others, vandalism, and self-aggression, which are often induced by psychotic symptoms and environmental factors [1]. The incidence of aggressive behavior in patients with schizophrenia is the highest among all types of mental disorders [2]. Previous studies have shown that approximately 87.8% of individuals with mental disorder-induced aggressive behavior have been diagnosed with schizophrenia [3, 4]. The aggressive tendencies of patients with schizophrenia are a result of various complex factors. Based on the social information processing model, external stimuli interpretation and processing considerably affect the behavior of an individual [5]. These factors can be categorized as historical factors (such as childhood experiences), patient factors (including gender, age, self-esteem, and psychological resilience), and clinical factors (such as disease characteristics). The recurrence of aggressive behavior not only poses a great threat to the stability of the pateint’s family and social harmony but also severely affects the prognosis and recovery of the patient. Therefore, focusing on aggressive behavior in patients with schizophrenia and understanding the risk factors for such behavior is indispensable for providing effective prevention and early intervention strategies.

Childhood trauma refers to the traumatic experiences that occur during the childhood and adolescence period of the patient. Studies have shown that childhood trauma incidence rates among people diagnosed with schizophrenia are notably higher compared to the General population [6]. Childhood traumatic experiences are important factors that affect the aggression of the individual and help predict antisocial behavior and personality [7, 8]. Yin Yan [9] reported how childhood trauma affects aggressive behavior in patients with schizophrenia, with increased trauma exposure amplifying such behavior. Studies have indicated that patients with schizophrenia have low self-esteem, self-esteem and aggressive behavior have a negative connection in patients with schizophrenia [10]. Social bond theory indicates that low self-esteem can damage social bonds, leading to inappropriate behavior and restricting the ability of the individual to handle social issues. Recurring frustrations often prompt aggressive behavior induced by psychotic symptoms, social biases, and other psychological factors [11]. Psychological resilience is a valuable personality trait that increases adaptability and recovery capabilities, providing patients with schizophrenia with the means to manage their illness and gain insight into its complexities. With increased psychological resilience, mental disorders may slow or reverse their progression [12]. Scientific findings suggest that aggressive behavior and psychological resilience have a negative connection in patients with schizophrenia [13]. Therefore, childhood trauma, self-esteem, and psychological resilience are important factors affecting aggressive behavior in patients with schizophrenia.

In previous studies, the risk factors for aggressive behavior in patients with schizophrenia were usually screened using logistic regression analysis [14, 15], which may be inadequate for an intuitive and efficient risk assessment for aggressive behavior. The aforementioned limitations can be addressed through Nomogram. Nomogram visualize regression results based on multifactor regression analysis and are often used for joint diagnosis of multiple indicators, predicting certain clinical outcomes, or the probability of specific events. This study aimed to apply Nomogram to the predictive research of aggressive behavior in patients with schizophrenia. We constructed a prediction model to obtain the probability of aggressive behavior, making the results more readable and facilitating the identification of high-risk populations.

## Materials and methods

### Design

In this study, patients with schizophrenia who met the inclusion criteria were enrolled based on convenience sampling from two psychiatric specialty hospitals in Daqing City and Chifeng City between March 2021 and July 2023. Patients with schizophrenia from Daqing Municipal Third Hospital were placed in the training set, and patients with schizophrenia from Chifeng Municipal Anding Hospital were placed in the validation set. The training set was used for model development and internal validation, whereas the validation set was used for external validation of the model. Patients enrolled in this study were divided into aggressive and non-aggressive groups based on whether there was at least one obvious and recorded personal attack episode (including obvious wounding and self-injurious behavior) following diagnosis.

### Participants

Diagnostic interviews in this study were conducted independently by two deputy chief physician using the International Classification of Diseases-10 diagnosis criteria. Inclusion criteria: ① patients who met the International Classification of Diseases-10 diagnosis criteria for schizophrenia; ② patients who are 18–65 years old; ③ patients who achieved the stable period through oral antipsychotic drug treatment, as judged by the following criteria: delusion, hallucinatory behaviors, exaggeration, and suspicion/victimization items in the positive and negative symptom scale (PANSS), abnormal thought content scores of ≤ 5 in the general psychopathology scale, and PANSS conceptual disorganization scores of ≤ 4, if all the aforementioned criteria were met, the patient was considered to be in a stable period of schizophrenia [16]; ④ patients with partial insight (PANSS G12 score < 4) [17]; ⑤ patients possessing communication and comprehension abilities, and those who could complete the given assessment; and ⑥ patients who voluntarily participated in this study. Exclusion criteria: patients with a coexisting history of mental developmental delay, dementia, and other mental disorders or those who were accompanied by severe physical illnesses. Ethical approval was obtained from the Ethics Committee of Harbin Medical University, and it conformed to the ethical guidelines of the Helsinki Declaration. All enrolled patients signed informed consent.

Information was gathered through direct encounters. Before conducting the survey, investigators received identical training-covering topics, including survey objectives, communication languages, potential obstacles during data collection along with remedies for such problems, and on-the-spot verification procedures for questionnaire legitimacy. Before filling out the questionnaires, comprehensive guidance on completion protocols was provided to ensure informed participation from the study sample. Following the on-site validations, questionnaires were considered complete and were logically organized after the survey.

### Measures

#### General information questionnaire

The general information questionnaire designed by the research team, includes gender, education level, marital status, number of recurrence, age of onset, age, duration of disease.

#### Positive and Negative Symptom Scale (PANSS)

The questionnaire consisted of 33 items and had a seven-level scoring system. Assessment indicators included the total score ranging from 30 to 210, positive and negative symptom scale scores ranging from 7 to 49, general psychopathology scale score ranging from 16 to 112. Higher scores on PANSS total score and various subscales indicate more severe clinical symptoms [16, 17]. The internal consistency reliability was 0.87 (Cronbach’s a). The structure, validity and reliability of PANSS (Chinese version) are acceptable [18]. The assessment was performed by psychiatric doctors to assess the presence and severity of psychiatric symptoms in patients with schizophrenia. The scale’s Cronbach’s α coefficient in this study was measured to be 0.887.

#### The Childhood Trauma Questionnaire-Short Form(CTQ-SF)

Comprising 28 items, this scale assessed five types of neglect or abuse (including emotional neglect, physical neglect, emotional abuse, physical abuse, and sexual abuse). Each item was rated on a five-point scale, with each subscale score ranging from 5 to 25, and the total score ranging from 25 to 125. Additionally, three items were used for validatory assessment. The reliability and validity of this scale have been verified in the Chinese population with schizophrenia. The Cronbach’s α coefficient of the Chinese CTQ-SF was 0.81, and the two-week re-test reliability was 0.81 (*P* < 0.01) [19]. The scale’s Cronbach’s α coefficient in this study was measured to be 0.990.

#### Connor- Davidson resilience scale(CD-RISC)

In 2003, Conner and Davidson [20] developed a scale consisting of 25 items. Each item was rated on a Likert five-level scale, with the total score ranging from 0 to 100. Higher scores indicated better psychological resilience. The scale was translated and revised by Yu Xiaonan et al. in 2007 and adjusted to the following three dimensions: resilience (13 items), strength (8 items), and optimism (4 items). The internal consistency of the Chinese version was 0.91, indicating that the questionnaire was suitable for the Chinese population [21]. The scale’s Cronbach’s α coefficient in this study was measured to be 0.962.

#### Self-esteem scale(SES)

In 1965, Rosenberg developed a scale designed to assess overall feelings of self-worth and self-acceptance in adolescent individuals [22]. The scale consisted of 10 items, with higher scores indicating higher levels of self-esteem. The scale commonly used for measuring the self-esteem.

of patients with severe mental disorders. This scale was translated and validated in Chinese [23], and its internal consistency was excellent Cronbach’s α coefficient was 0.82 [24]. The scale’s Cronbach’s α coefficient in this study was measured to be 0.971.

### Statistical analysis

Data analyses were conducted using the Statistical Package for the Social Sciences 25.0 and R 4.3.1 (packages such as glm, predict, rms, and DynNom were used). Binary logistic regression was performed for variable selection and model construction. The optimal threshold for the prediction model was calculated based on the Youden index, and Nomogram was plotted. Bootstrap resampling was performed 1,000 times for internal validation, and the validation set data were used for external validation. The discriminative ability of the model was evaluated using the C-statistic (C > 0.7 indicating good discriminative ability), and model calibration was assessed through the calibration curves and Brier scores (scores < 0.25 were preferred). The significance level was set at α = 0.05.

## Results

### Participant characteristics

A total of 429 patients were included in this study, of which 113 (113/429, 26.34%) showed aggressive behavior and 316 (316/429, 73.66%) did not showed aggressive behavior. There were significant differences in age (*p* < 0.001) and duration of disease (*p* = 0.001) between the aggressive and non-aggressive groups. The basic information of the two groups of patients is shown in Table 1.

**Table 1:** Comparison of general demographic characteristics of patients with or without Aggressive Behavior

Variables	Aggressive behavior group (*n* = 113, %)	Non-aggressive behavior group (*n* = 316, %)	*t*/*χ*^*2*^	*P value*
Gender			0.060^a^	0.807
Male	68(60.18%)	186(58.86%)		
Female	45(39.82%)	130(41.14%)		
Education level			0.836^a^	0.841
Primary school	14(12.39%)	32(10.13%)		
Junior middle school	20(17.70%)	50(15.82%)		
High school	47(41.59%)	143(45.25%)		
Junior college and above	32(28.32%)	91(28.80%)		
Marital status			0.182^a^	0.980
Married	45(39.82%)	132(41.77%)		
Divorced	29(25.66%)	81(25.63%)		
Unmarried	38(33.63%)	100(31.65%)		
Widowed	1(0.89%)	3(0.95%)		
Number of recurrence			2.250^a^	0.134
≤ 3	34(30.09%)	120(37.97)		
>3	79(69.91%)	196(62.03%)		
Age of onset			1.371^a^	0.242
≤ 30	68(60.18%)	170(53.80%)		
>30	45(39.82%)	146(46.20%)		
Age	35.46 ± 9.256	38.96 ± 8.350	3.710^b^	<**0.001**
Duration of disease	9.34 ± 5.598	11.49 ± 5.643	3.485^b^	**0.001**

^a^is the χ^2^ test

^b^is the t test

### Analysis of influencing factors of aggressive behavior

The presence or absence of aggressive behavior (assigned values: 1 = aggressive behavior and 0 = no aggressive behavior) was used as the dependent variable, and age, duration of illness, childhood trauma score, self-esteem score, resilience score, PANSS score, positive and negative symptom scale scores, and general psychotic symptom scale score were included as independent variables for the binary logistic regression analysis. The results showed that the model incorporated the following six factors: age, duration of illness, childhood trauma score, self-esteem score, psychological resilience score, and positive-symptom scale score (Table 2). The area under the receiver operating characteristic curve was 0.790, and the χ^2^ value of 5.218 was obtained from the Hosmer–Lemeshow test (*p* = 0.734), indicating a good model fit. Based on the Youden index, the optimal threshold for the constructed model was calculated to be 0.308, with a sensitivity of 70.0%, specificity of 81.4%, and accuracy of 83.4%. A younger age, shorter duration of illness, lower self-esteem score, lower psychological resilience score, higher childhood trauma score, and higher positive-symptom scale score were associated with a higher likelihood of exhibiting aggressive behavior.

**Table 2:** Logistic regression analysis results of influence factors for aggressive behavior in patients with schizophrenia

Variables	*B*	*SE*	*Wald*	*OR*(95% *CI*)	*P value*
Age	−0.043	0.015	8.056	0.958(0.930,0.987)	**0.005**
Duration of disease	−0.057	0.024	5.733	0.945(0.902,0.990)	**0.017**
Childhood trauma	0.035	0.008	19.428	1.035(1.020,1.052)	**0.000**
Self-esteem	−0.034	0.017	4.133	0.966(0.935,0.999)	**0.042**
Resilience	−0.035	0.007	23.507	0.966(0.952,0.979)	**0.000**
Positive symptom	0.151	0.056	7.430	1.164(1.043,1.297)	**0.006**
Negative symptom	0.069	0.059	1.369	1.072(0.954,1.203)	0.242
General pathological symptom	0.100	0.057	3.102	1.105(0.989,1.234)	0.078
PANSS scores	−0.092	0.056	2.743	0.912(0.817,1.017)	0.098

### Construction of the Nomogram to predict the risk of aggressive behavior and assess model performance

**Fig. 1:**
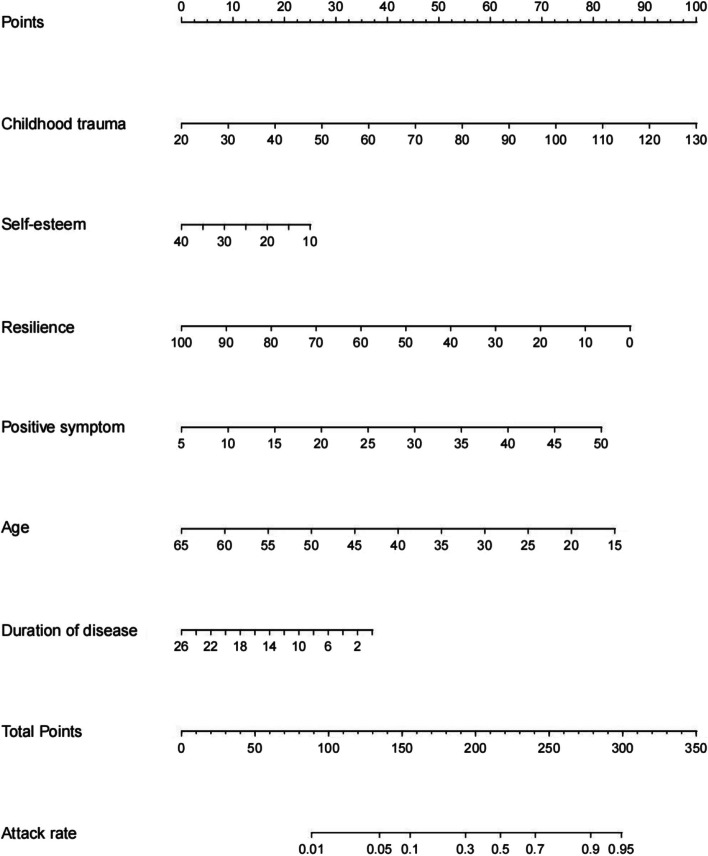
Nomogram for predicting aggressive behavior risk in stable patients with schizophrenia

**Fig. 2:**
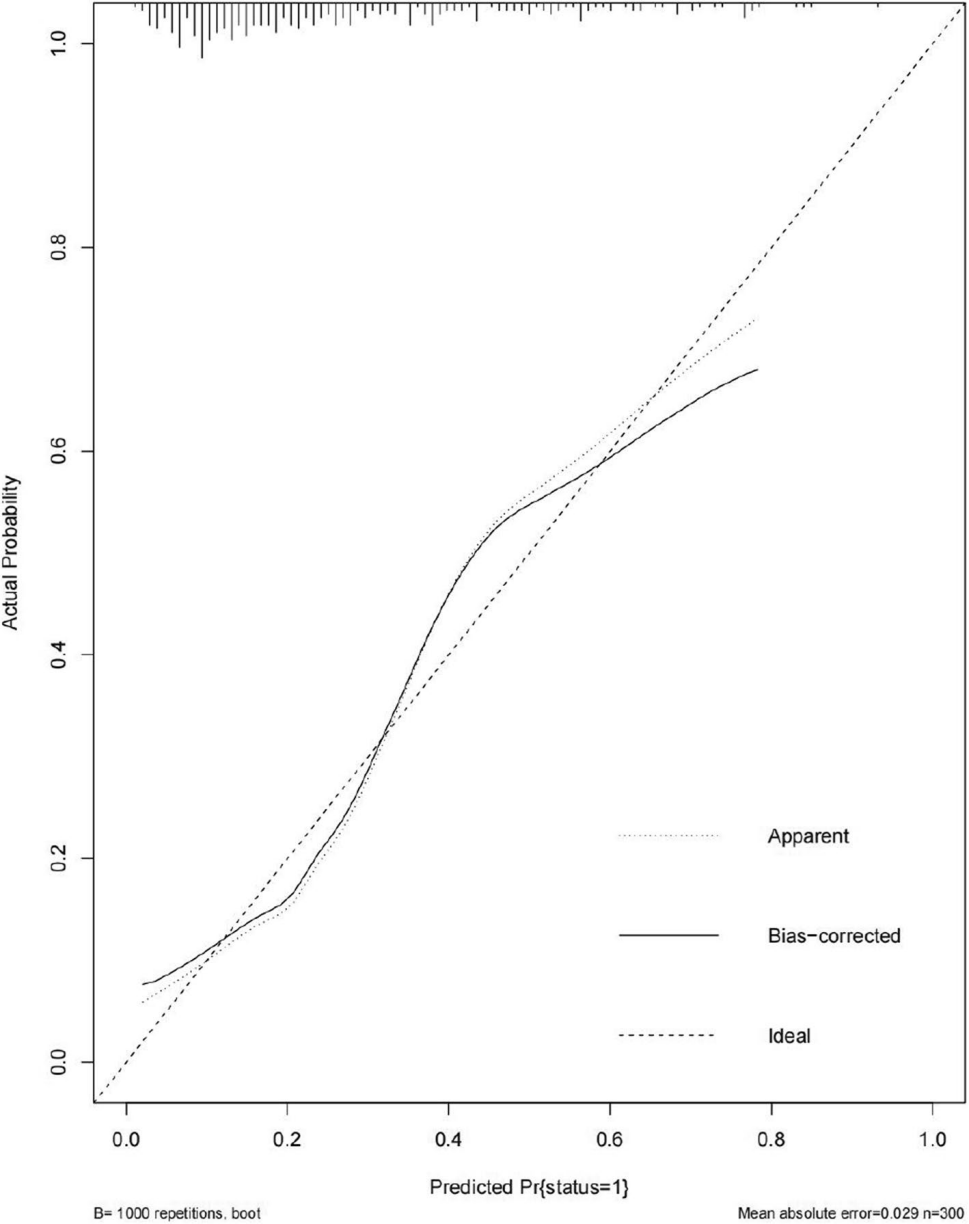
The calibration curve of model internal validation

**Fig. 3:**
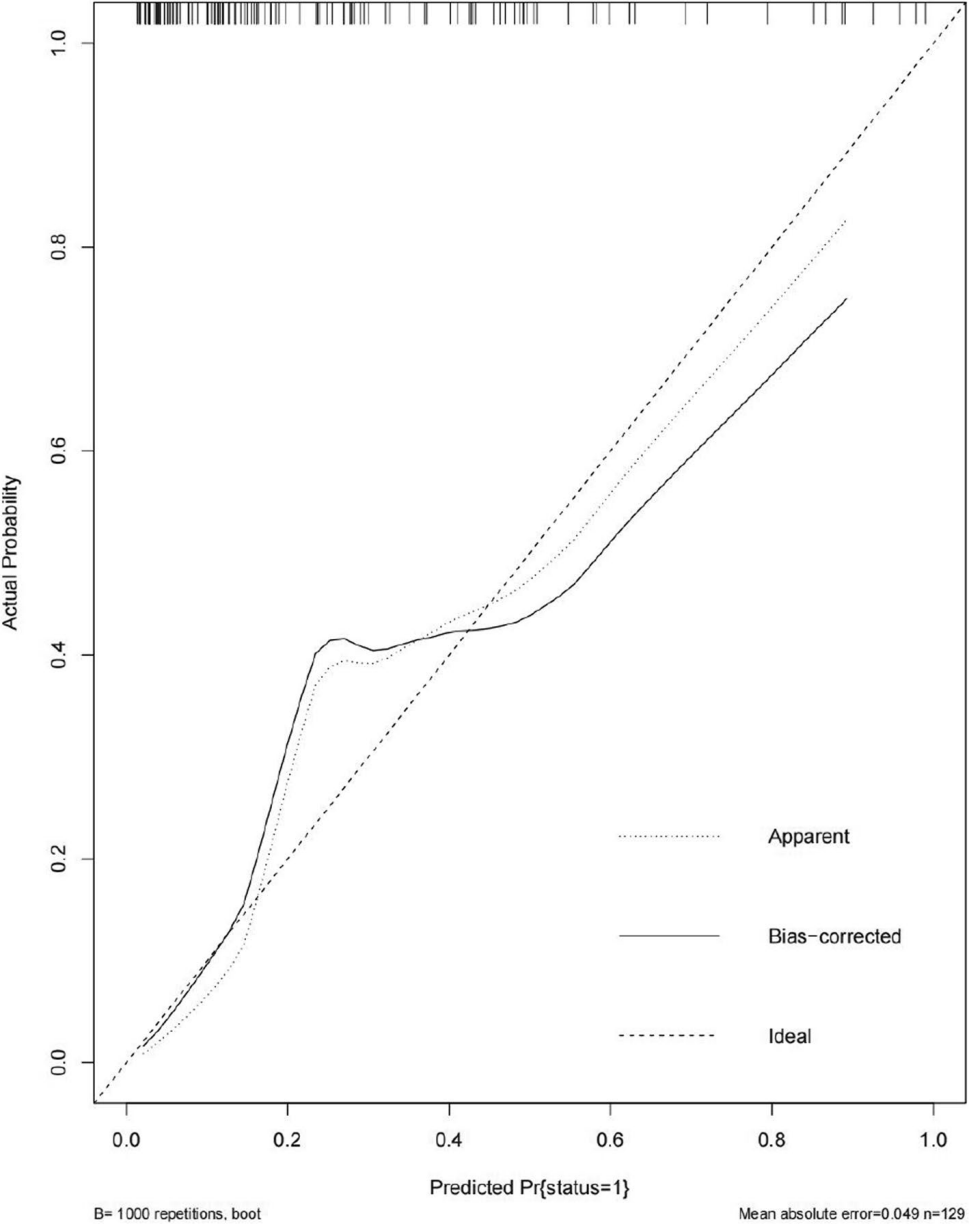
The calibration curve of model external validation

An Nomogram was constructed depicting the risk of aggressive behavior occurrence in patients with schizophrenia based on the six independent predictive factors selected via binary logistic regression analysis (Fig. 1). Each predictive factor had a corresponding score on the upper scale. The total score of the six factors in the model was projected onto the “risk of aggressive behavior” axis, which represented the predicted probability of aggression occurrence. A higher value indicated a higher risk of aggressive behavior. Internal validation was performed using the training set through bootstrap resampling 1,000 times. The constructed model exhibited good discrimination and calibration in predicting the risk of aggressive behavior occurrence. The C-statistic was 0.759, and the calibration curve showed a high consistency between the predicted risk by the model and the actual outcomes, The Brier score was 0.151 (Fig. 2). External validation was performed using the data from the validation set comprising 129 cases, and the results showed good discrimination and calibration. The C-statistic was 0.819, which indicated a good consistency between the predicted risk and actual outcomes, The Brier score was 0.134 (Fig. 3).

## Discussion

Herein, the detection rate of aggressive behavior in patients with schizophrenia was 26.34%, which was consistent with a meta-analysis study that showed that the incidence of aggressive behavior ranges from 15.3 to 53.2% [25]. Aggressive behavior inflicts severe harm on the families of patients, society, and healthcare personnel and hinders social and occupational rehabilitation of the patient [26], which leads to increased treatment costs and hospitalization time [27]. Therefore, constructing a prediction model for predicting the risk of aggressive behavior is crucial for early identification and intervention. Herein, we constructed a visual Nomogram prediction model with the risk predicting abilities of 0.759 and 0.819 in the training and validation sets, respectively. The calibration curve showed no significant difference between the predicted and measured values, indicating the sufficient discriminatory ability and clinical utility of the model.

The results of this study indicated that patients with aggressive behavior were younger and had shorter illness duration compared with those of patients without aggressive behavior. Age and duration of disease were the predictive risk factors for aggressive behavior in patients with schizophrenia, which was consistent with previous research findings [28–30]. However, some studies have shown no correlation between aggressive behavior in patients with schizophrenia and general demographic data such as age and duration of disease [25]. This may be because, with the increased age and extended duration of disease, most patients develop a certain understanding of the disease and handle issues with more maturity and stability, allowing them to effectively control their behavior. The inconsistency in results might be attributed to regional and cultural differences and variations in sample selection and size.

The results of this study showed that aggressive behavior were positively correlated with positive symptoms and childhood traumas in patients with schizophrenia, corroborated by prior investigations [31, 32]. Per the assessment, positive manifestations in patients with schizophrenia included delusions, muddled thinking, bizarre behavior, and cognitive inconsistencies, all of which greatly affect how they interpret reality, resulting in extreme frustration, varying mood swings, increased arousal levels, and challenges in controlling actions, ultimately leading to aggressive behavior [33]. To effectively handle the threat of aggressive behavior, healthcare workers must carefully evaluate the level of intensity among positively symptomatic patients with schizophrenia during the initial stages of treatment and proactively intervene accordingly. Studies show that approximately 80.67% of patients with schizophrenia endured childhood traumas [34, 35]. Childhood trauma experiences may affect the development of specific neural circuits and structures in the developing brain, leading to atypical development of the hypothalamic–pituitary–adrenal axis, which may result in emotional regulatory dysfunction and an increased risk of aggressive behavior in those who develop schizophrenia. Hence, clinical attention should be paid to the effects of childhood trauma on aggressive behavior in patients with schizophrenia and help medical personnel manage their emotions and behaviors while ensuring the safety of the patient.

The study findings suggest that aggressive behavior were negatively correlated with self-esteem and psychological resilience, which is consistent with the findings of previous studies [36]. Self-esteem is affected by interpersonal relationships, and people with schizophrenia tend to shut themselves off because of their long-term illness, so they are likely to choose aggressive behavior when taking actions to avoid interpersonal failure and emotional regulation dysfunction [37]. Therefore, improving the self-esteem of patients with schizophrenia is a crucial part of intervention to reduce the occurrence of aggressive behavior. Psychological resilience is a crucial variable in positive psychology, and higher psychological resilience effectively negates the effect of stress events, allowing individuals to maintain relatively good stress susceptibility. This is consistent with the protective mechanism of psychological resilience reported in previous schizophrenia studies. In the biopsychological model of psychological resilience, the core of psychological resilience involves appropriate stress responses and rapid recovery after stress exposure. Previous studies have shown that social adaptation training and mindfulness-based stress reduction training can improve psychological resilience in patients with schizophrenia [38, 39]. Therefore, improving the psychological resilience of patients is of immense significance in decreasing the susceptibility to stress and the occurrence of aggressive behavior. In addition, studies have shown that psychological resilience has a positive impact on self-esteem. When the level of psychological resilience of patients is low, the occurrence of aggressive behavior can be reduced by improving the level of self-esteem of patients [40].

### Limitations

This study has some limitations that should be addressed. First, the data collected using the childhood trauma scale can be subjected to recall bias. Second, the sample size of this study was designed to fulfill research requirements and could be expanded in future studies for further validation of the model. Furthermore, in this study, only the influential factors of aggressive behavior were evaluated, and psychotherapy and counseling intervention was not used to evaluate the possible protective factors in the evaluation process. Lastly, the study samples were only obtained from two psychiatric hospitals in China, and the applicability of the results to other countries or regions needs further verification in multicenter studies.

## Conclusion

In this study, we constructed an nomogram model that quantitatively scores age, duration of disease, positive symptoms, childhood trauma, self-esteem, and resilience. A higher total score indicates a higher risk of aggressive behavior occurrence. Psychiatric professionals can assign scores based on relevant risk factors in patients with schizophrenia, intuitively assessing the risk of aggressive behavior occurrence and providing early attention and intervention for the high-risk population.

## Data Availability

Due to the privacy of the participants involved in the study data, the datasets generated and/or analyzed in the study are not currently publicly available but are available from the corresponding authors of this study upon reasonable request.
